# Prevalence of metabolic syndrome in Saudi Arabia - a cross sectional study

**DOI:** 10.1186/s12902-018-0244-4

**Published:** 2018-03-05

**Authors:** Khalid Al-Rubeaan, Nahla Bawazeer, Yousuf Al Farsi, Amira M. Youssef, Abdulrahman A. Al-Yahya, Hamid AlQumaidi, Basim M. Al-Malki, Khalid A. Naji, Khalid Al-Shehri, Fahd I. Al Rumaih

**Affiliations:** 1 University Diabetes Center, College of Medicine, King Saud University, PO Box 18397, Riyadh, Riyadh 11415 Saudi Arabia; 2 Nutrition Department, University Diabetes Center, King Saud University, Riyadh, Saudi Arabia; 3 Registry Department, University Diabetes Center, King Saud University, Riyadh, Saudi Arabia; 4 College of Medicine, King Saud University, Riyadh, Saudi Arabia

**Keywords:** Metabolic syndrome, Prevalence, Risk factors, Obesity, Diabetes, Cross-sectional survey, Saudi Arabia

## Abstract

**Background:**

The evaluation of metabolic syndrome in a society predisposed to the diabetes mellitus epidemic opens a new avenue to understanding this rapidly growing global metabolic problem. Although Saudi Arabia reports one of the highest prevalence levels of obesity and diabetes, a very limited number of epidemiological studies have examined the prevalence of metabolic syndrome. Therefore, the main aim of the current study was to estimate the prevalence of metabolic syndrome and its risk factors among the adult Saudi population in comparison to other countries.

**Methods:**

A total of 12,126 Saudi subjects were randomly recruited from the 13 administrative regions, and evaluated for metabolic syndrome and its risk factors. This exercise was carried out by trained physicians, through clinical evaluations and overnight fasting blood glucose and lipid profile measurements. Both the International Diabetes Federation (IDF) and modified National Cholesterol Education Program and Adult Treatment Panel III (NCEP ATP III) Criteria were employed, and subjects with metabolic syndrome were identified using country-specific waist circumference cutoff values.

**Results:**

The prevalence of metabolic syndrome in Saudi Arabia was found to be 39.8% (34.4% in men and 29.2% in women) and 31.6% (45.0% in men and 35.4% in women), according to the NCEP ATP III and IDF criteria, respectively. Metabolic syndrome was also observed to be more prevalent among men and older subjects. The most frequently observed component of metabolic syndrome was found to be low levels of high-density lipoprotein (HDL), followed by abdominal obesity. The most significant risk factors in the studied cohort included age ≥ 45, smoking history, low educational level, and living in urban areas.

**Conclusions:**

This study shows a high prevalence of metabolic syndrome in Saudi Arabia, and thereby warrants urgent implementation of preventive health care strategies to reduce both morbidity and mortality related to this medical problem.

## Background

Metabolic syndrome was first recognized by the medical community during the late 1980s and was characterized by the clustering of abdominal obesity, elevated blood pressure, hyperglycemia, and dyslipidemia [1]. This syndrome has been redefined through several amendments by different scientific bodies, and was finally defined by either the ATP III [2] or IDF criteria [3], wherein the IDF criteria mandates the presence of central obesity as one of the components of metabolic syndrome. Subjects with metabolic syndrome are at increased risk for coronary heart disease (CHD), and the presence of metabolic syndrome alone can predict approximately 25% of all new-onset cardiovascular disease (CVD) [4]. In addition, metabolic syndrome is associated with an increased risk of death from CHD, CVD, and all other causes [5]. It affects nearly one quarter of the adult population worldwide, and its prevalence varies, according to the definition used, ethnicity under study, and level of urbanization [6]. Among the most recent studies, the prevalence of metabolic syndrome has been reported to be between 10% and 84% worldwide depending on the age, sex, and ethnicity of the population [7]. The National Health and Nutrition Examination Survey (NHANES), using the ATP III criteria, showed the prevalence of metabolic syndrome to be 34.5%, whereas this figure was 39.0% with the IDF criteria [8]. These findings are different from those observed in an Irish study that reported a prevalence at 21.4% and 13.2%, using the IDF and ATP III definitions, respectively [9]. The prevalence was even lower among Chinese individuals, reported at 7.9% and 15.1% using ATP III and IDF definitions, respectively [10].

The Middle East and North African (MENA) region is known for its high prevalence of metabolic syndrome, where it has been reported to be 45.5% and 24.3% in Tunisia, using the IDF criteria and ATP III definition, respectively [11]. Gulf countries, being part of the Middle East, have shown a prevalence of metabolic syndrome that ranges from 17% in Oman [12] to 40.5% in the United Arab Emirates (UAE) [13], according to the ATP III and IDF criteria, respectively. Although no recent nationwide survey has evaluated the prevalence of metabolic syndrome in Saudi Arabia, Al-Nozha et al. [14] reported it to be 39.3% in 2005, using the 2001 ATP III criteria.

This study is a part of the Saudi Abnormal Glucose Metabolism and Diabetes Impact Study (SAUDI-DM) [15] that investigates the prevalence of metabolic syndrome and its risk factors in the adult Saudi society, in comparison to other societies.

## Methods

### Subjects

**Fig. 1:**
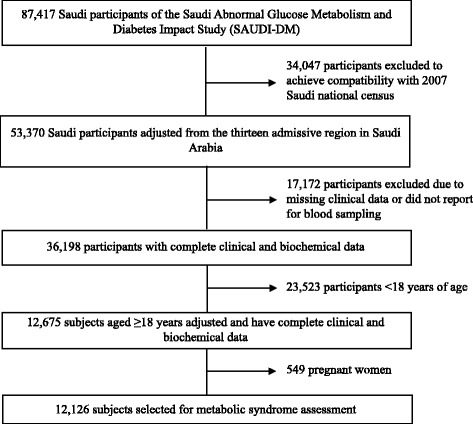
Flow chart of the study cohort selection

The SAUDI-DM is a nationwide, household cross-sectional population-based survey that uses a multistage stratified cluster sampling technique. The study recruited 87,417 Saudi nationals between 2007 and 2009 from the 13 administrative regions of Saudi Arabia. The data of all study participants were adjusted for age, area of residency (urban and rural, according to the definitions of the Ministry of Municipal and Rural Affairs), and sex distribution, using the Saudi national census for the year 2007 that led to the exclusion of 34,047 non-compatible participants [15]. For the current analysis, we further excluded 17,172 subjects with incomplete clinical data, or those who did not report for blood sampling. Subjects younger than 18 years of age (totaling 23,523) were also excluded. A total of 549 women from this cohort were found to be pregnant and had to be excluded. The final study cohort comprised of 12,126 Saudi subjects aged ≥18 years, with complete clinical and biochemical data, as shown in Fig. 1.

The current study was conducted by trained physicians and nurses, through primary healthcare centers, to secure accurate and complete data. The data that were collected consisted of general demographic and clinical information including age, sex, highest level of education attained, and monthly income, in addition to history of diabetes, hypertension, and dyslipidemia. The SAUDI-DM study was reviewed and approved by the Institutional Review Board at the College of Medicine, King Saud University.

### Anthropometric measurements and vital signs

Anthropometric measurements, including weight, height, and waist circumference, were taken with the subjects in a standing position, wearing light clothing without shoes. Weight and height were assessed, using a weighing scale (Adam Equipment Oxford CT USA, model MDW-250 L) with a capacity of 250 kg and reliability of 0.1 kg. Waist circumference was measured at the midpoint between the top of the iliac crest and the lower margin of the last palpable rib. Hip circumference was measured at the widest part of the body below the waist. The waist-to-hip ratio (WHR) was calculated by dividing the waist circumference by the hip circumference. Systolic (SBP) and diastolic blood pressure (DBP) measurements were taken from the left arm, after at least 5 min of rest, with the subjects in a sitting position, using a standardized mercury sphygmomanometer (Baumanometer, Model 0320, W.A. Baum Co., Inc. USA).

### Laboratory analysis

All subjects were asked to report to the nearest primary health care center (PHCC) after more than 10 h of overnight fasting, after which 10 mL of venous blood was collected using a sodium fluoride tube. All blood samples were sent to the central laboratory at the Strategic Center for Diabetes Research in the Riyadh, the capital city of the Kingdom, using portable refrigerators in which the temperature was maintained between 4 °C and 8 °C. Plasma was stored at − 20 °C at the central laboratory. The blood glucose assessment was conducted, using the glucose oxidase/peroxidase method; whereas blood cholesterol was measured using the esterase oxidase/peroxidase method; and levels of high-density lipoprotein (HDL), low-density lipoprotein (LDL), and triglycerides were determined, using the glycerokinase oxidase/peroxidase method.

### Definition of metabolic syndrome

Metabolic syndrome was defined, using both the modified National Cholesterol Program Adult Treatment Panel III (NCEP ATP III) and the International Diabetes Federation (IDF) criteria, and implementing the new cutoff value for waist circumference in Saudi society [16]. Therefore, subjects were considered to have metabolic syndrome if they had central obesity that was defined by a waist circumference ≥ 92 cm in men and ≥87 cm in women, along with two or more of the following criteria, as per the IDF definition [3]: high fasting glucose level ≥ 100 mg/dL (5.6 mmol/L), or patients known to have diabetes mellitus and/or on treatment for diabetes; hypertriglyceridemia - serum triglyceride level ≥ 150 mg/dL (1.7 mmol/L); low HDL cholesterol - serum HDL cholesterol < 40 mg/dL (1.0 mmol/L) in men and < 50 mg/dL (1.3 mmol/L) in women, or patients known to have dyslipidiema; high blood pressure - SBP ≥ 130 mmHg and/or DBP ≥ 85 mmHg, or patients known to have hypertension, and/or on treatment for hypertension. The NCEP-APT III criteria for metabolic syndrome were met if an individual had three or more of the aforementioned criteria [4].

### Statistical analysis

Data were analyzed using the SPSS statistical package version 21. Continuous variables were expressed as mean ± standard deviation (SD), and categorical variables were expressed as percentages. The *t*- test was used for continuous variables and chi-squared test for categorical variables. Risk factors for metabolic syndrome were assessed using univariate, age- and sex-adjusted, and multivariate logistic regression models. The odds ratio and 95% confidence intervals were used to express different risk factors. A *p*-value less than 0.05 was used as the level of significance.

## Results

The studied cohort of 12,126 subjects represents the Saudi population over 10-year age intervals, with a mean age of 35.7 ± 15.0 years, wherein men were significantly older than women, and both had similar distribution. More subjects lived in urban areas than in rural areas. The prevalence of obesity, particularly as morbid obesity (body mass index (BMI) ≥ 30 kg/m2), was higher among women versus men [(36.5% versus 29.4% (*p* < 0.001)]. Men had a significantly higher mean waist circumference; whereas women had a higher mean hip circumference. The mean WHR was significantly higher among men. Only 20.7% of the study cohort had a relatively high monthly income (> 8000 Saudi Riyals [SR]) and a higher proportion of men were smokers in comparison to women. Men had a significantly higher mean SBP and DBP, as well as higher mean fasting plasma glucose (FPG), mean LDL, and triglycerides. In contrast, mean HDL cholesterol was significantly higher among women. The prevalence of metabolic syndrome according to the IDF criteria was 31.6%; specifically, 34.4% in men and 29.2% in women. However, according to the ATP III criteria, the prevalence of metabolic syndrome was higher at 39.9%; specifically, 45.0% in men and 35.4% in women, as shown in Table 1.

**Table 1:** Baseline characteristics of the study cohort and the calculated metabolic syndrome prevalence

Total 12,126	Men 5571(45.94)		Women 6555(54.06)	*P* value
Discreptive analysis; mean (± SD)				
Mean age (years)	35.7 (±15.0)	36.1 (±15.2)	35.5 (±14.8)	*P* value
Mean WC (cm)	87.0 (±16.7)	89.71 ± 17.11	84.75 ± 16.03	0.035
Mean Hip (cm)	99.1 (±16.8)	98.7 (±16.8)	99.4(±16.8)	< 0.001
Mean W-H ratio	0.9 (±0.12)	0.9(±0.1)	0.9 (±0.1)	0.014
Mean Systolic Bp (mmHg)	117.7 (±13.8)	119.7 (±13.1)	116.0 (±14.1)	< 0.001
Mean Diastolic Bp (mmHg)	76.1 (±8.6)	77.3 (±8.9)	75.2 (±8.7)	< 0.001
Mean FPG (mmol/L)	5.7 (±2.4)	5.8 (±2.6)	5.6 (±2.3)	< 0.001
Mean LDL Cholesterol (mmol/L)	3.2 (±1.1)	3.2 (±1.1)	3.2 (±1.0)	< 0.001
Mean triglyceride (mmol/L)	1.6 (±1.2)	1.8 (±1.3)	1.5 (±0.1)	0.012
Mean HDL cholesterol (mmol/L)	0.1 (±0.3)	0.9 (±0.3)	1.0 (±0.3)	< 0.001
Frequancy anlysis; number (%)				
Age groups: 18-29 years	5196 (42.9)	2328(41.8)	2868(43.8)	< 0.001
30-39 years	2660 (21.9)	1276(22.9)	1384(21.1)	
40-49 years	2207 (18.2)	942(16.9)	1265(19.3)	
50-59 years	1086 (8.1)	524(9.4)	562(8.6)	
60-69 years	573(4.7)	315(5.7)	258(3.9)	
≥ 70 years	404(3.3)	186(3.3)	218(3.3)	
BMI groups < 18.5	673(5.6)	315(5.7)	358(5.5)	< 0.001
18.5-24.9	3783(31.2)	1807(32.4)	1976(30.1)	
25-29.9	3642(30.0)	1812(32.5)	1830(27.9)	
≥ 30	4028(33.2)	1637(29.4)	2391(36.5)	
Monthly Income < 4000 SR	5255(43.3)	2175(39.0)	3080(46.1)	< 0.001
4000-8000 SR	4438(36.6)	2173(39.0)	2265(34.6)	
> 8000 SR	2433(20.1)	1223(21.95)	1210(18.46)	
Smoking	1564(12.9)	1475(26.5)	89(1.4)	< 0.001
Educational level: Illiterate	2417(19.9)	554(9.94)	1863(28.42)	
Less than high school	3648(30.1)	1855(33.3)	1793(27.4)	
More than or equal high school	6061(49.1)	3162(56.8)	2899(44.2)	
Family history of: Diabetes Mellitus	6200(51.1)	2896(51.1)	3304(50.4)	0.083
Hypertension	4212(34.7)	1887(33.9)	2325(35.5)	0.066
Metabolic Syndrome Prevalence				
IDF criteria (WC + ≥ 2risk factors)	3833(31.6)	1917(34.4)	1916(29.2)	< 0.001
NCEP-ATP-III criteria (3 or more risk factors)	4828(39.8)	2507(45.0)	2321(35.4)	< 0.001

NCEP-ATP-III;, National Cholesterol Education Program and Adult Treatment Panel III, HDL; high density lipoprotein, IDF; International Diabetes Federation, LDL; low density lipoprotein, WC; waist circumference, WHR; waist-to-hip ratio

The prevalence of metabolic syndrome and its components increased with age, except in the age group ≥70 years. The most frequently observed component of metabolic syndrome was low HDL that affected around 80% of the sample. Abdominal obesity ranged between 25% and 70%, whereas elevated blood glucose affected 25% to 60% according to the age group. Elevated triglycerides and high blood pressure were the components of metabolic syndrome that occurred least frequently.

**Fig. 2:**
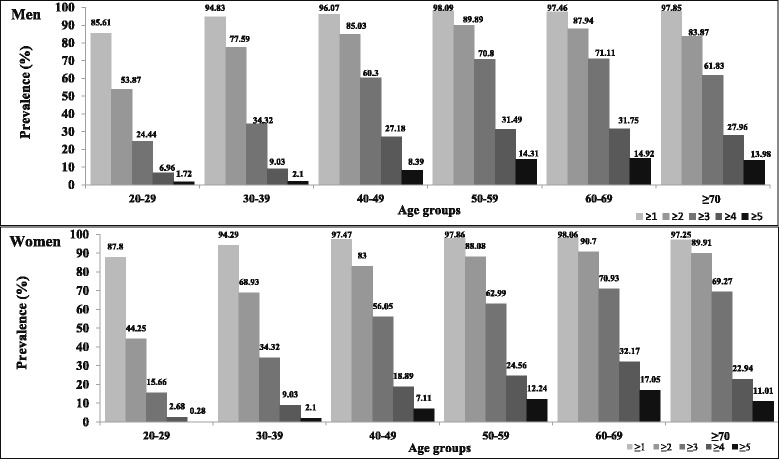
Relative frequency of one or more components of metabolic syndrome, according to different ages and sex distribution

Both male and female subjects showed an increasing prevalence of metabolic syndrome with age, although this was more pronounced according to the ATP III criteria. Men had a higher prevalence of metabolic syndrome compared to women in the younger age groups; whereas women had a higher prevalence in the age group ≥70 years. Middle-aged men and women had an almost similar prevalence of metabolic syndrome. Women in different age groups showed a high prevalence of low HDL and abdominal obesity, whereas the prevalence of elevated blood pressure, blood glucose, and triglycerides was higher among men as compared to women in 10-year age intervals, as shown in Table 2. Figure 2 shows the frequency of one or more components of metabolic syndrome, according to differences in age and sex distribution. As the number of metabolic syndrome components increase, the relative frequency is reduced, regardless of age group or sex. The frequency of three or more components of metabolic syndrome increased with age in both male and female subjects. In addition, the frequency of three or more risk factors for metabolic syndrome was found to be higher among men than women, with the exception of the > 70 age group, in which women had a higher frequency than men.

**Table 2:** Prevalence of metabolic syndrome (95%CI) and its components according to age and sex strata

Age groups	Abdominal obesity	Elevated blood pressure	Elevated blood glucose	Elevated triglycerides	Low HDL cholesterol	Metabolic syndrome	
						IDF	NCEP-ATP III
Total							
18-29 years	25.3(24.1-26.5)	12.0 (11.2-12.9)	25.0(23.8-26.2)	22.0(20.85-23.11)	75.7 (74.5-76.9)	13.4(12.4-14.3)	19.6(18.5-20.7)
30-39 years	51.4(49.5-53.3)	24.7(23.0-26.3)	34.6(32.7-36.4)	38.83(37.0-40.7)	80.2(78.6-81.7)	33.8(31.96-35.6)	42.7(40.8-44.6)
40-49 years	66.3(64.3-68.3)	38.6(36.5-40.6)	46.8(44.7-48.8)	41.55(39.5-43.6)	81.4(79.8-83.0)	49.4(47.3-51.5)	57.9(55.8-59.9)
50-59 years	70.4(67.7-73.2)	53.31(50.3-56.3)	56.5(53.6-59.5)	42.4(39.4-45.3)	82.2(79.96-84.5)	56.3(53.3-49.2)	66.8(64.0-69.6)
60-69 years	69.8(66.1-73.5)	63.4(59.4-67.3)	59.9(55.9-63.9)	44.0(39.9-48.0)	79.8(76.5-83.1)	58.5(54.4-62.5)	71.0(67.3-74.7)
≥ 70 years	59.7(54.9-64.4)	66.8(62.2-71.4)	57.7(52.9-62.5)	38.4(33.6-43.1)	78.7(74.7-82.7)	50.7 (45.9-55.6)	65.8(61.2-70.5)
Men							
18-29 years	27.7(25.9-29.5)	16.5(15.0-18.0)	28.4(26.6-30.3)	28.4(26.6-30.2)	71.0(69.2-72.9)	16.4(14.9-17.9)	24.4(22.7-26.2)
30-39 years	52.4(49.6-55.1)	30.4 (27.9-32.9)	41.4(38.7-44.1)	49.61(49.9-52.4)	77.6(75.3-79.9)	39.26(36.7-41.94)	51.7(49.0-54.5)
40-49 years	63.1(60.0-66.1)	41.2(38.1-44.3)	49.2(46.0-52.3)	51.1(47.9-54.3)	77.3(74.6-80.0)	49.5(46.3-52.7)	60.3(57.2-63.4)
50-59 years	68.9(64.9-72.9)	58.2(54.0-62.4)	57.4(53.2-61.7)	49.6(45.3-53.9)	79.8(76.3-83.2)	57.25(53.0-61.5)	70.8(66.9-74.7)
60-69 years	69.2(64.1-74.3)	61.9(56.5-67.3)	58.7(53.3-64.2)	47.0(41.5-52.5)	75.2(70.5-80.0)	58.7(53.3-64.2)	71.11(66.1-76.1)
≥ 70 years	55.9(48.8-63.1)	66.7(59.9-73.4)	59.7(52.6-66.7)	43.0(35.9-50.1)	69.4(62.7-76.0)	45.2(38.0-52.3)	61.8(54.9-68.8)
Women							
18-29 years	23.3(21.7-24.8)	8.40(7.38-9.42)	22.18(20.7-23.7)	16.77(15.4-18.1)	79.53(78.1-81.0)	10.9(9.8-12.1)	15.7(14.3-17.0)
30-39 years	50.5(47.9-53.1)	19.4(17.3-21.4)	28.3(25.9-30.6)	28.9(26.5-31.3)	82.5(80.5-84.5)	28.7(26.3-31.5	34.3(31.8-36.8)
40-49 years	68.7(66.1-71.3)	36.6(34.0-39.3)	45.0(42.2-47.7)	34.5(31.9-37.1)	84.5(82.5-86.5)	49.3(46.6-52.1)	56.1(53.3-58.8)
50-59 years	71.9(68.2-75.6)	48.8(44.6-52.9)	55.7(51.6-58.8)	35.6(31.6-39.6)	84.5(81.5-87.5)	55.3(51.2-59.5)	63.0(59.0-67.0)
60-69 years	70.5(65.0-76.1)	65.1(59.3-70.9)	61.2(55.3-67.2)	40.3(34.3-46.3)	85.3(81.0-89.6)	58.1(52.1-64.2)	70.9(65.4-76.5)
≥ 70 years	62.8(56.4-69.3)	67.0(60.7-73.2)	56.0(49.4-62.6)	34.4(28.1-40.7)	86.7(82.2-91.2)	55.5(48.9-62.1)	69.27(63.15-75.39)

NCEP-ATP-III;, National Cholesterol Education Program and Adult Treatment Panel III, HDL; high density lipoprotein, IDF; International Diabetes Federation, LDL; low density lipoprotein

### Risk factors

When the risk factors for metabolic syndrome were assessed in the current study, any age ≥ 45 years was the most important and significant risk factor in both unadjusted and multivariate models. The male gender, smoking, and increased BMI were each independently and significantly associated with an increased risk of metabolic syndrome. Higher monthly income and low educational level were found to be significant risk factors for metabolic syndrome, when the unadjusted model was used. However, high monthly income remained significant only in the age- and sex-adjusted model, and low educational level remained independently significant only in the multivariate adjusted model. Living in an urban area was significantly associated with an increased risk of metabolic syndrome in the age- and sex- or multivariate adjusted models. Family history of DM and hypertension were also associated with an increased risk of metabolic syndrome after adjusting for age and sex, whereas only family history of hypertension remained significant in the multivariate adjusted model, as shown in Table 3.

**Table 3:** Risk factors for metabolic syndrome odds ratio (95% CI) assessment with sex and gender and multivariate adjustment

Number of components factors	Unadjusted OR (95% CI)	Age and sex Adjusted OR (95% CI)	Multivariate OR (95% CI)
Age ≥ 45 years	4.4(4.0-4.8)	–	3.9(3.4-4.5)
Male gender	1.5(1.39-1.60)	–	2.0(1.8-2.3)
Smoking	1.6(1.4-1.8)	1.2(1.0-1.4)	1.4(1.1-1.6)
High monthly income	1.1(1.0-1.2)	1.2(1.1-1.3)	1.1(1.0-1.2)
Low educational level	1.5(1.4-1.7)	1.1(1.0-1.2)	1.3(1.1-1.5)
Urban residency	1.0(1.0-1.1)	1.2(1.0-1.2)	1.1(1.0-1.3)
Body mass index	1.6(1.1-1.2)	1.1 (1.13-1.15)	1.2(1.1-1.2)
Family history of diabetes mellitus	1.0(1.0-1.1)	1.3(1.2-1.4)	1.0(0.9-1.1)
Family history of hypertension	1.0(1.0-1.1)	1.3(1.2-1.4)	1.2(1.1-1.4)

OR; odds ratio, CI; confidence interval. Adjustement was performed for all factors listed in the table

## Discussion

Saudi Arabia is known to be one of the top countries worldwide with a high prevalence of diabetes, and similarly high rate of obesity that has a direct effect on more than one third of its adult population [17]. In addition, the prevalence of other components of metabolic syndrome is reaching soaring heights in the Kingdom [14]. Therefore, with such a high prevalence of the various components of metabolic syndrome, the prevalence of metabolic syndrome in Saudi Arabia would be expected to exceed that is reported in other countries. The current study shows the prevalence of metabolic syndrome in Saudi Arabia to be 39.8% according to the ATP III criteria and 31.6% according to the IDF criteria, when local waist circumference cutoff values have been implemented [16]. Gulf countries that have passed through similar socio-economic transitions have also shown similar levels of prevalence of metabolic syndrome, in spite of the use of lower cutoff values for waist circumference in both men and women in the current study [18–20]. The prevalence of metabolic syndrome in these countries ranged from 33.7% [21] to 40.5% according to the IDF criteria [13], and from 17% [12] to 39.6% [11] according to the ATP III criteria. The prevalence of metabolic syndrome reported in other MENA countries show a comparatively lower prevalence. The prevalence in Iran was reportedly 32.1% and 33.2% in 2006, according to the IDF and ATP III criteria, respectively [22]; and that in Tunisia it was 30.0% according to the ATP III criteria [23].

These findings indicate that in terms of the prevalence of metabolic syndrome, Saudi Arabia is one of the leading MENA countries. The prevalence of metabolic syndrome among the Saudi population is also higher than that reported among ethnicities, such as the adult Spanish [24] and Australian [25] populations, in which the prevalence is reported as 31.0% for Spaniards and 30.7% for Australians, according to the ATP III and IDF criteria, respectively. In addition, the prevalence of metabolic syndrome in the Saudi population, according to the revised ATP III criteria, was higher than that reported in Korea and South Asia [26, 27], despite of the use of lower Asian-specific cutoff values for abdominal obesity of 90 cm and 80 cm for men and women, respectively.

The current study shows that men were more frequently affected by metabolic syndrome than women, based on both sets of criteria. These findings are inconsistent with those reported among the Caucasian ethnicity [28]. The male predominance observed in the current study could be explained by the higher frequency of diabetes, hypertension, hypertriglyceridemia, and smoking among men in Saudi society, as compared to other ethnicities [15, 29–31]. Furthermore, the waist circumference cutoff values that were used for men in the community under study were lower than those proposed by the ATP III and IDF [2, 3]. However, this was not the case for women, as the waist circumference cutoff values used for Saudi women were higher than those specified by the IDF criteria, and closer to those of the ATP III criteria [16]. Another reason behind the low prevalence of metabolic syndrome among women in Saudi society is the lower rate of smoking among Saudi women. This protects them from the negative effects of tobacco smoking on the emergence of several metabolic disorders, including the more serious insulin resistance, hyperinsulinemia, and increased waist circumference [32].

Women in this cohort, older than 70 years of age, had a higher prevalence of metabolic syndrome than men. This could be explained on one hand by the post-menopausal estrogen withdrawal effect that increases the prevalence of chronic diseases [33], and on the other hand by the poor survival observed among men with metabolic syndrome at a younger age. In addition, this study highlighted the fact that being male was a significant and independent risk factor for metabolic syndrome, until the age of 70 years.

Similar to the observations reported in the NHANES study [34], the prevalence of metabolic syndrome in the current study increased with age, reaching its peak in the sixth and the seventh decades, and decreased thereafter. This might be because age is associated with hormonal alterations, increased visceral obesity, and insulin resistance [35]. Another explanation for such age-dependent increases in the prevalence of metabolic syndrome is the parallel increase in the prevalence of the distinct components of metabolic syndrome, mainly diabetes and hypertension, with age in the Saudi population [15, 29]. In addition, the current study shows that age is a significant and independent risk factor for metabolic syndrome.

Low HDL cholesterol was the most frequent component of metabolic syndrome observed in the current study, and this finding has also been reported in other population-based studies in South Asia [26] and the Middle East [22, 36]. Low HDL cholesterol was observed more frequently in women; a finding that is consistent with most of the other studies conducted among different ethnicities [22, 36]. This observation could be explained by the higher rate of abdominal obesity observed among women in the current study, a factor that is known to lower HDL values [37]. In the present cohort, 43.4% of the participants had more than two risk factors for metabolic syndrome, a number that is higher than that observed among Omanis [38], but lower than that observed among Kuwaitis [36]. These subjects represent a high-risk group for the development of metabolic syndrome. This warrants early intervention to prevent the progression of this very expensive and even life-threatening syndrome, by adopting alternative measures that include lifestyle modifications.

Living in urban areas and a lower education level were significant risk factors for metabolic syndrome in Saudi society, a finding that is similar to those observed in other ethnicities [39–41]. This significant association is expected, because urbanization is associated with an increased prevalence of cardiovascular risk factors, such as hypertension, obesity, and dyslipidemia, as it offers economic improvement to the rural population and exposes them to additional health risks, including a poor diet and sedentary lifestyle [40, 41]. Such effects of urbanization are obvious in populations that have experienced rapid urbanization and swift lifestyle changes, such as those in Saudi Arabia and other Gulf countries [42]. The significant association between a low educational level and metabolic syndrome could be mediated by other risk factors, such as smoking and high carbohydrate intake [23].

No significant effect of a high monthly income on metabolic syndrome was noted in the current study. This finding was unexpected and differed from previous reports of other Gulf countries; however, it is in line with the inconsistency observed in the reported relationship between a high-income status and the development of metabolic syndrome [7, 20].

The current study gains its strength from the fact that it was a nationwide study with a large number of participants. Another strength of the current study was the use of a clear case definition that was based on diagnostic confirmation, using blood tests to identify diabetic and dyslipidemic cases, and country-specific waist circumference cutoff values. However, the study was limited by the fact that it was a cross-sectional study; thus, the causal relationship between metabolic syndrome and certain risk factors could not be elicited. The study was also compromised by the exclusion of physical activity and dietary assessments, both of which are important contributing factors for metabolic syndrome.

## Conclusions

In conclusion, this study places Saudi Arabia as one of the countries with the highest prevalence of metabolic syndrome. Although the risk factors for metabolic syndrome in Saudi society were similar to those reported internationally, men were particularly at a greater risk of having metabolic syndrome. A high income had no effect on the prevalence of metabolic syndrome; thus, any prevention program should not consider income as a selection factor.

These findings are startling and should alert policy makers in Saudi Arabia to consider the implementation of preventive lifestyle interventions that include smoking cessation and weight control programs. Furthermore, in order to prevent metabolic syndrome, policy makers should consider the promotion of a healthy diet and physical activity in the planning of future health care strategies in Saudi Arabia.

## Abbreviations

BMI: Body mass index
CHD: Coronary heart disease
CI: Confidence intervals
DBP: Diastolic blood pressure
FPG: Fasting plasma glucose
HDL: High-density lipoprotein
IDF: International Diabetes Federation
IRB: Institutional Review Board
LDL: Low-density lipoprotein
MENA: Middle East and North Africa
NCEP ATP III: National Cholesterol Education Program and Adult Treatment Panel III
NHANES: National Health and Nutrition Examination Survey
OR: Odds ratio
PHCC: Primary health care center
SAUDI-DM: Saudi Abnormal Glucose Metabolism and Diabetes Impact Study
SBP: Systolic blood pressure
SD: Standard deviation
UAE: United Arab Emirates
WHR: Waist-to-hip ratio
